# Tristetraprolin Inhibits Poly(A)-Tail Synthesis in Nuclear mRNA that Contains AU-Rich Elements by Interacting with Poly(A)-Binding Protein Nuclear 1

**DOI:** 10.1371/journal.pone.0041313

**Published:** 2012-07-26

**Authors:** Yu-Lun Su, Shun-Chang Wang, Pei-Yu Chiang, Nien-Yi Lin, Yu-Fang Shen, Geen-Dong Chang, Ching-Jin Chang

**Affiliations:** 1 Graduate Institute of Biochemical Sciences, College of Life Science, National Taiwan University, Taipei, Taiwan; 2 Institute of Biological Chemistry, Academia Sinica, Taipei, Taiwan; Wayne State University, United States of America

## Abstract

**Background:**

Tristetraprolin binds mRNA AU-rich elements and thereby facilitates the destabilization of mature mRNA in the cytosol.

**Methodology/Principal Findings:**

To understand how tristetraprolin mechanistically functions, we biopanned with a phage-display library for proteins that interact with tristetraprolin and retrieved, among others, a fragment of poly(A)-binding protein nuclear 1, which assists in the 3'-polyadenylation of mRNA by binding to immature poly(A) tails and thereby increases the activity of poly(A) polymerase, which is directly responsible for polyadenylation. The tristetraprolin/poly(A)-binding protein nuclear 1 interaction was characterized using tristetraprolin and poly(A)-binding protein nuclear 1 deletion mutants in pull-down and co-immunoprecipitation assays. Tristetraprolin interacted with the carboxyl-terminal region of poly(A)-binding protein nuclear 1 via its tandem zinc finger domain and another region. Although tristetraprolin and poly(A)-binding protein nuclear 1 are located in both the cytoplasm and the nucleus, they interacted *in vivo* in only the nucleus. *In vitro*, tristetraprolin bound both poly(A)-binding protein nuclear 1 and poly(A) polymerase and thereby inhibited polyadenylation of AU-rich element–containing mRNAs encoding tumor necrosis factor α, GM-CSF, and interleukin-10. A tandem zinc finger domain–deleted tristetraprolin mutant was a less effective inhibitor. Expression of a tristetraprolin mutant restricted to the nucleus resulted in downregulation of an AU-rich element–containing tumor necrosis factor α/luciferase mRNA construct.

**Conclusion/Significance:**

In addition to its known cytosolic mRNA–degrading function, tristetraprolin inhibits poly(A) tail synthesis by interacting with poly(A)-binding protein nuclear 1 in the nucleus to regulate expression of AU-rich element–containing mRNA.

## Introduction

Tristetraprolin (TTP) binds AU-rich elements (AREs) in the 3′-untranslated region (3′-UTR) of short-lived, mature, cytosolic mRNA [1]–[3]. TTP recognizes AREs via its tandem zinc finger (TZF) domain, and its binding causes targeted mRNAs to be rapidly deadenylated and then further degraded [4], [5]. TTP directs targeted mRNAs to the ARE-mediated mRNA decay machinery by interacting with its components [6]. The 5′ to 3′ degradation of mRNA occurs at processing bodies [7], which include Dcp1a, Dcp2, Edc3, Xrn1, Ago2, and Ago3 that interact with TTP [8]–[10]. TTP also interacts with PM-Scl75 and Rrp44, two subunits of the cytosolic exosome, in which 3' to 5' mRNA degradation occurs [10], [11]. TTP can also associate with the Ccr4-Caf1-Not deadenylation complex and thereby promotes deadenylation of targeted mRNAs [12]–[14]. Because all known TTP-interacting proteins are components of large complexes, it is not clear which of these components directly interact with TTP.

In serum-deprived NIH/3T3 cells, 70% of total TTP was found in the nucleus and, within 5 min of serum addition, ∼80% of the TTP translocated into the cytoplasm [15]. Approximately 80% and 20% of expressed His-tagged TTP in human embryonic kidney (HEK)293T cells are cytoplasmic and nuclear in nature, respectively [16]. MAPKAP kinase 2 and p38 MAP kinase phosphorylate TTP [17], [18] and, by doing so, reduce its mRNA-destabilizing activity [19] and direct it from the nucleus into the cytoplasm [20], [21]. TTP destabilization of mRNA seems to occur only in the cytoplasm, even though TTP shuttles between the nucleus and the cytoplasm [22]. In mammals, how TTP functions in the nucleus is unclear. In yeast, Cth2, a TTP ortholog, modulates the selection of the poly(A) site in nuclear ARE-containing mRNA and thereby produces unstable, extended transcripts [23].

Although many proteins have been co-immunoprecipitated with TTP, how they interact among themselves is still unclear. For the study reported herein, to find proteins that directly interact with TTP, we performed a phage-display biopan using TTP as bait. Some of the in-frame cDNA sequences that encode the phage library protein fragments that bound TTP (Table S1) are those for poly(A)-binding protein nuclear 1 (PABPN1), which binds to poly(A) tails of nuclear pre-mRNAs, facilitates poly(A) elongation, and defines the lengths of newly synthesized poly(A) tails [24], [25]. Poly(A) tails are synthesized by poly(A) polymerase (PAP), but its activity requires the cleavage and polyadenylation specificity factor (CPSF) to first cleave the 3′-signaling region of the pre-mRNA. Only then, in conjunction with CPSF and PABPN1, can PAP synthesize a poly(A) tail of defined length, i.e., ∼250 nucleotides in mammals [26], [27].

Having found that a PABPN1 fragment was capable of interacting with TTP, we then confirmed that TTP and PABPN1 directly interact using *in vitro* pull-down assays that incorporated recombinant proteins. Next, the interacting TTP and PABPN1 domains were identified. We discovered that TTP also directly interacts with PAP and inhibits the PABPN1-assisted PAP polyadenylation of ARE-containing mRNA. A TTP construct restricted to the nucleus suppressed TNFα ARE-mediated luciferase activity.

## Results

### Biopanning with a Phage-display Library for TTP-interacting Proteins

**Figure 1:**
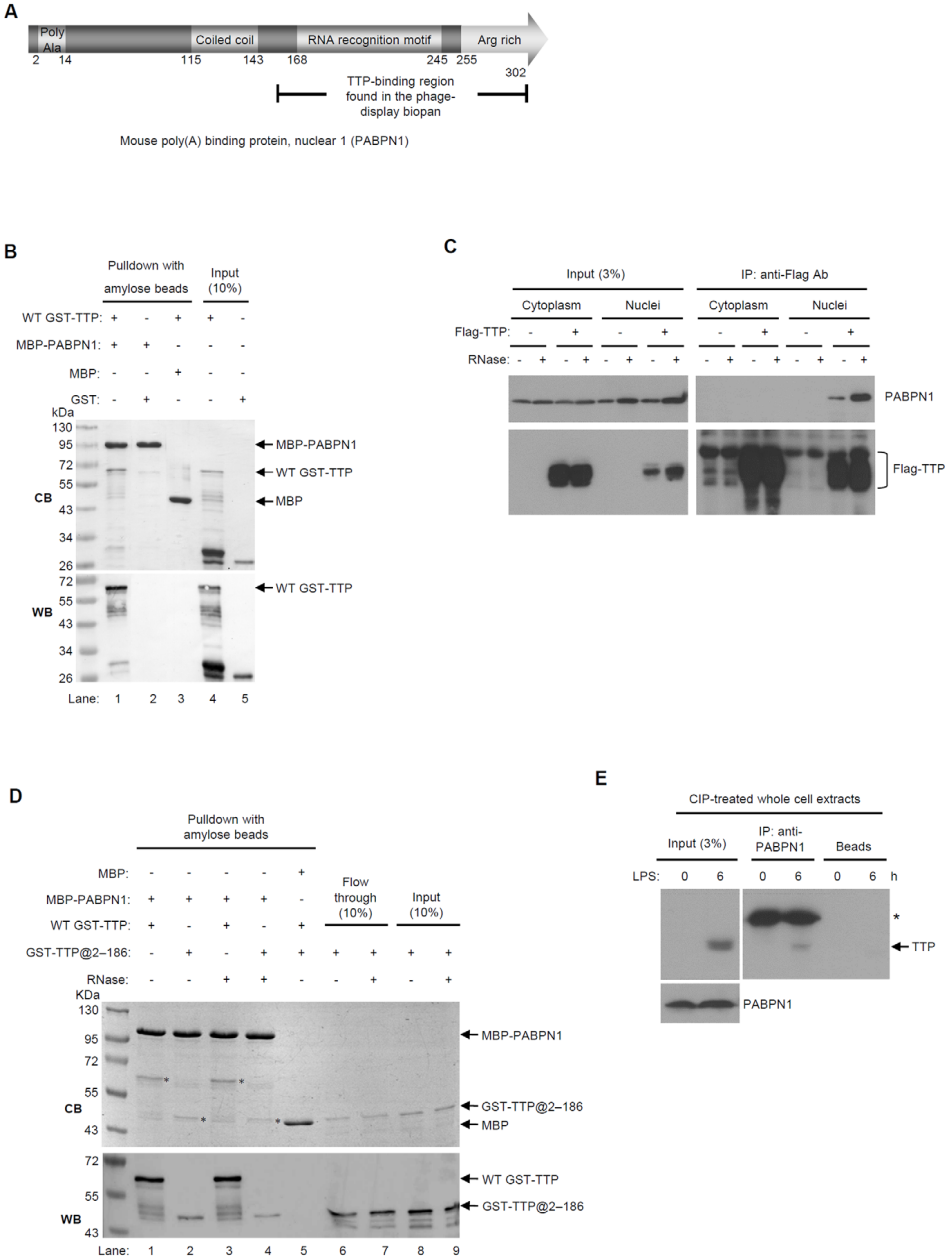
PABPN1 and TTP interact. (A) Schematic of the domain structure of mouse PABPN1. (B) Pull-down assay of MBP-PABPN1 and WT GST-TTP. Bait MBP-PABPN1 or MBP (control) was immobilized on amylose resin and WT GST-TTP or GST (control) was then added. After washing extensively, the pulled-down protein complexes were separated by SDS-PAGE and visualized with Coomassie Blue (CB, upper panel), and western blotted with anti-GST (WB, lower panel). The lanes labeled “10% input” correspond to samples that each contained 10% of the protein used for the pull-down experiments. (C) Co-immunoprecipitation of endogenous PABPN1 by Flag-TTP. HEK293T cells (2×10^6^) were transfected with 10 µg of pCMV-Flag-TTP or a control vector, and the isolated cytosolic and nuclear extracts digested with (+) or without (-) RNase were immunoprecipitated with anti-Flag M2 agarose resin. The complexes pulled down were separated by SDS-PAGE, western blotted, and probed with anti-Flag or anti-PABPN1 antibodies as indicated. The lanes labeled “Input (3%)” represent 3% of the protein used for the co-immunoprecipitation. (D) A substantial amount of GST-TTP was pull-down by MBP-PABPN1 after RNase treatment. 1 µg of each protein was used per pull-down assay. Before the pull-down step, 12 µg RNase A and 200 units of RNase T1 were added into each reaction mixture to digest any RNA present. The gels show that both GST-TTP constructs were precipitated by MBP-PABPN1 after RNase treatment. The asterisks indicate the positions of WT GST-TTP and GST-TTP@2-186. (E) Endogenous TTP and PABPN1 interact in RAW264.7 cells. Cells were untreated or induced with 100 ng/ml LPS for 6 h and were then harvested and lysed to obtain whole cellular protein extracts. Before co-immunoprecipitation, cellular lysates were treated with calf intestinal phosphatase (CIP) for 90 min. Co-immunoprecipitation was performed using PABPN1-conjugated protein A Sepharose. Interaction of TTP and PABPN1 was detected with anti-TTP (arrow). The asterisk indicates the position of the IgG heavy chain. The signal for immunoprecipitated PABPN1 was hard to discriminate from that of the heavy chain. Only the input PABPN1 protein is displayed (lower left panel).

To find proteins that directly interact with TTP, we used a high-throughput T7 phage library for biopanning. The library was created using cDNA from RAW264.7 cells. Purified maltose-binding protein-TTP-(His)_6_ (MBP-TTP-(His)_6_) was immobilized on Ni-NTA resin, and then the phage library was added. The phages that bound to the resin were then recovered and plated. A total of 408 clones from the fourth, ninth, and tenth biopannings were sequenced, and their sequences were subjected to a Blast search of the National Center for Biotechnology Information mouse gene database. Among the 408 clonal sequences, 46 were in-frame and represent five proteins (Table S1). One of the sequences is that for residues 131–302 of PABPN1, for which a complete domain schematic is shown in Fig. 1A. Residues 131–302 include the RNA recognition motif (RRM), which binds poly(A) sequences [28], [29], and the arginine-rich region, which directly interacts with PAP to stimulate its polyadenylation activity [27]. The frequency of clones retrieved by biopanning that encode a PABPN1 nucleotide sequence is quite large (Table S1). Because PABPN1 and TTP function in mRNA polyadenylation and deadenylation, respectively, we asked if TTP could affect polyadenylation by PABPN1.

### TTP Interacts with PABPN1

To confirm the results of the biopans, i.e., that TTP interacts with PABPN1, pull-down assays using purified N-terminally glutathione S-transferase-tagged wild-type (WT)TTP **(**GST-TTP) and MBP-PABPN1 were performed. The proteins pulled down were resolved by sodium dodecyl sulfate-polyacrylamide gel electrophoresis (SDS-PAGE), and anti-GST was used for western blotting. WT GST-TTP was pulled down by MBP-PABPN1, but not by MBP, and GST was not pulled down by MBP-PABPN1 (Fig. 1B, lower panel, lanes 1–3). The TTP/PABPN1 interaction also occurred in cells. Cytoplasmic and nuclear extracts from HEK293T cells that expressed Flag-TTP were immunoprecipitated with anti-Flag, and the ability of Flag-TTP to have interacted with PABPN1 *in vivo* was detected by western blotting with anti-PABPN1. PABPN1 interacted with Flag-TTP in the nucleus (Fig. 1C). Because both TTP and PABPN1 bind mRNA, we also assessed if the TTP/PABPN1 interaction depended on the presence of mRNA by adding RNase to the extracts to abolish any possible RNA-tethering effects. After RNase treatment, PABPN1 still co-immunoprecipitated with TTP in the nuclear extracts. Similar results were obtained with the *in vitro* pull-down assay (Fig. 1D). Therefore, TTP interacts with PABPN1 *in vitro* and in the nucleus, and in the absence of RNA, the TTP/PABPN1 interaction still can occur. Moreover, co-immunoprecipitation with cell extracts from LPS-stimulated RAW264.7 macrophages revealed that endogenous TTP indeed interacted with endogenous PABPN1 (Fig. 1E), indicating that TTP may participate in PABPN1-mediated reaction in cells.

### TTP Binds PABPN1 via its TZF Domain

**Figure 2:**
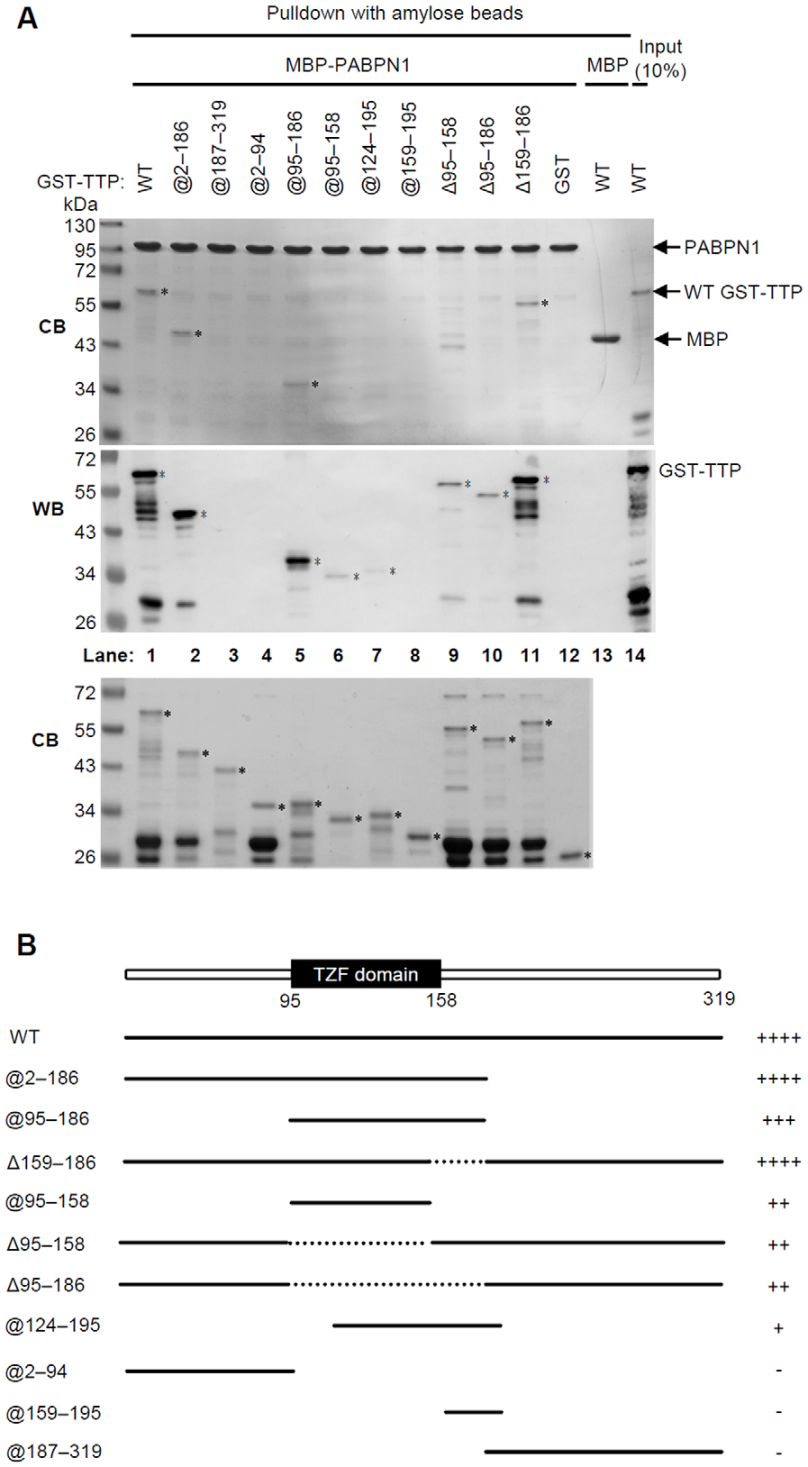
Identification of the TTP domain(s) that interact with PABPN1. (A) Pull-down assays for the GST-TTP deletion mutants by MBP-PABPN1. MBP-PABPN1 was immobilized on amylose resin and incubated with GST-TTP deletion mutants. The pulled-down proteins were subjected to SDS-PAGE and visualized with Coomassie Blue (CB, upper panel), and western blotted with anti-GST (WB, middle panel). Asterisks indicate the pulled-down TTP constructs. Lane 13: MBP was used as control bait for WT GST-TTP. The sample added to lane 14 was 10% that of the WT GST-TTP sample used in lane 1. (B) Schematics of the TTP constructs used in the pull-down assays of panel A. Included sequences are diagramed as heavy lines, and deleted regions are diagramed as dotted lines. Plus signs indicate the relative amount of a TTP construct pulled down by PABPN1, and a negative sign indicates that the TTP construct was not pulled down.

To identify which region(s) of TTP interacts with PABPN1, GST-TTP deletion mutants were expressed, purified, and used in pull-down assays. An SDS-PAGE gel that included all expressed GST-TTP constructs and was visualized with Coomassie Blue is shown in the lower panel of Fig. 2A. WT GST-TTP (@2-319), GST-TTP@2-186, and GST-TTP@95-186 interacted strongly with PABPN1, whereas the N-terminal GST-TTP@2-94 and the C-terminal GST-TTP@187-319 deletion mutants did not interact with PABPN1. (The numerical range following the @ sign indicates the included TTP sequence.) GST-TTP@95-158, which contains only the TZF domain, was stained more weakly than was GST-TTP@95-186 (Fig. 2A, lanes 1–6). Therefore, residues 159–186 of TTP**,** seemed to be important for PABPN1 binding, although GST-TTPΔ159–186 also bound PABPN1 (The numerical range that follows Δ identifies the region deleted from WT GST-TTP). GST was not pulled down by PABPN1, and MBP did not pull down WT GST-TTP. We thereby confirmed a direct interaction between TTP and PABPN1, and identified residues 95–186 of TTP as needed for tight TTP/PABPN1 binding. However, other regions in TTP also interacted with PABPN1 as weaker signals were found for GST-TTPΔ95–186 (Fig. 2A, lane 10). The relative binding abilities (as plus or negative signs) of the WT and mutant GST-TTPs with PABPN1 are diagramed in Fig. 2B.

### The C-terminal Region of PABPN1, which Contains the RRM and Arginine-rich Regions, is Important for the Binding of TTP

**Figure 3:**
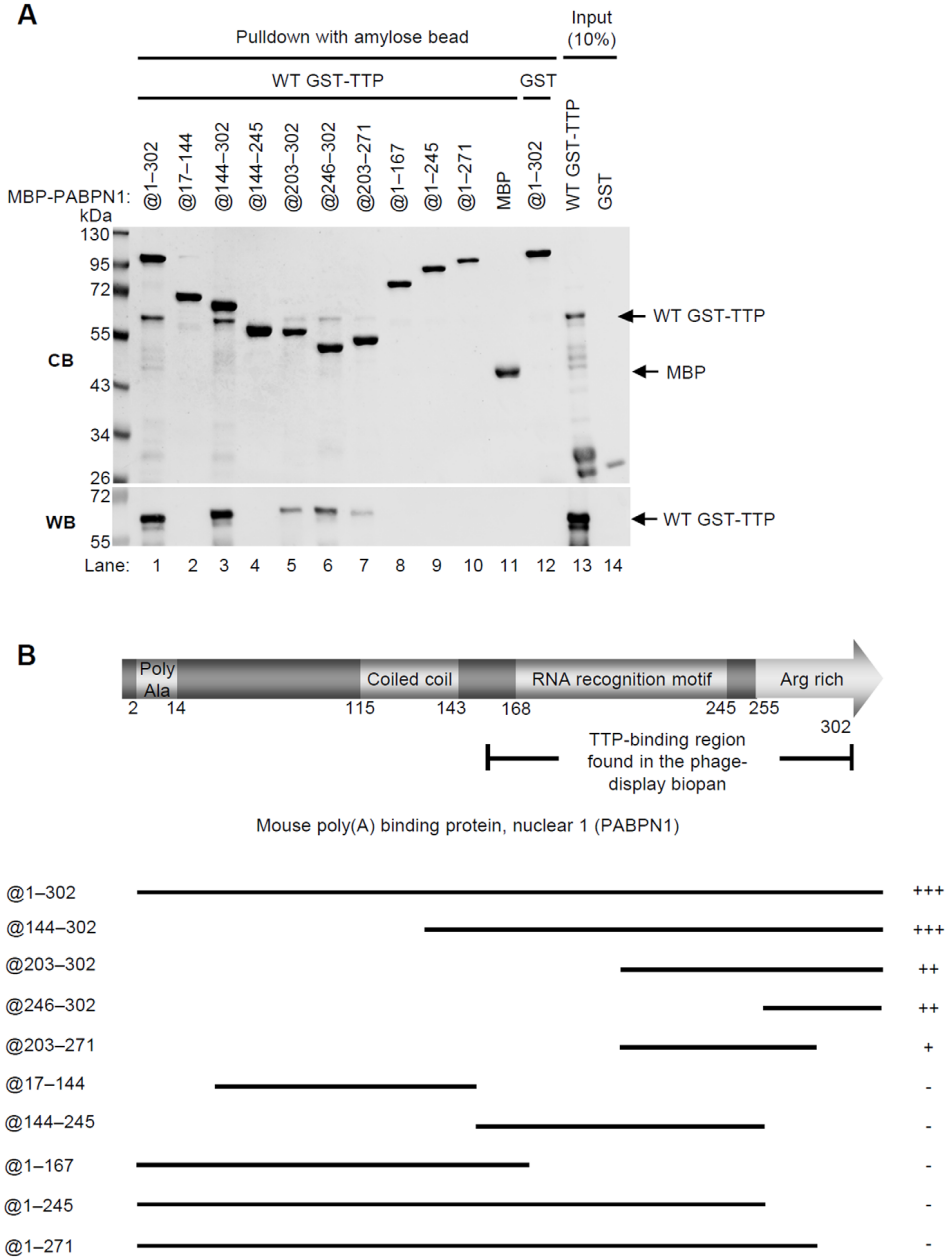
Identification of the PABPN1 domains that interact with TTP. (A) Pull-down assays using MBP-PABPN1 deletion mutations as bait for WT GST-TTP. Each MBP-PABPN1 deletion mutant was individually immobilized on amylose resin and then incubated with GST-TTP. After extensive washes, the pulled-down complexes were separated by SDS-PAGE and visualized with Coomassie Blue (CB), or western blotted with anti-GST (WB). GST incubated with MBP-PABPN1@1-302 served as the control (lane 12). (B) Schematics of the PABPN1 constructs used in the pull-down assays. Heavy lines identify the included sequences. Plus signs indicate the relative amount of GST-TTP pulled down by the PABPN1 constructs, and a negative sign indicates that GST-TTP was not pulled down.

PABPN1 can be divided into three parts: the N-terminal region, the RRM, and the C-terminal arginine-rich region, which is critical for PAP binding (Fig. 1A) [27]. Although we found by biopanning that a PABPN1 fragment containing the RRM and the arginine-rich region interacted with TTP, we wanted to more precisely define the region(s) that interacts with TTP because such knowledge would increase our understanding of how TTP affects mRNA metabolism in the nucleus. To define the TTP-interacting region of PABPN1, various MBP-PABPN1 deletion mutants were expressed, purified, and immobilized on amylose resin for use in pull-down assays with WT GST-TTP (Fig. 3A). MBP-PABPN1@1-302 and MBP-PABPN1@144-302 strongly interacted with WT GST-TTP (Fig. 3A, lanes 1 and 3). However, the C-terminal constructs MBP-PABPN1@203-302 and MBP-PABPN1@203-271, which each contain an incomplete RRM domain (Fig. 3A, lanes 5 and 7), and MBP-PABPN1@246-302, which does not contain an RRM domain (Fig. 3A, lane 6), bound WT GST-TTP less strongly. MBP-PABPN1@144-245, which contains only the RRM domain, did not bind TTP (Fig. 3A, lane 4) nor did MBP-PABPN1@17-144, MBP-PABPN1@1-167, MBP-PABPN1@1-271, and MBP-PABPN1@1-245 (Fig. 3A, lanes 2, 8–10). The relative binding abilities (as plus or negative signs) of the WT and mutant MBP-PABPN1 with WT GST-TTP are diagramed in Fig. 3B. Therefore, the RRM domain and the arginine-rich region are both required for full binding activity, which confirmed our biopanning results. Because the arginine-rich region has been shown to play an important role in the binding of PABPN1 to PAP, we hypothesized that TTP might compete with the PABPN1/PAP interaction.

### TTP binds PABPN1 and PAP Simultaneously

**Figure 4:**
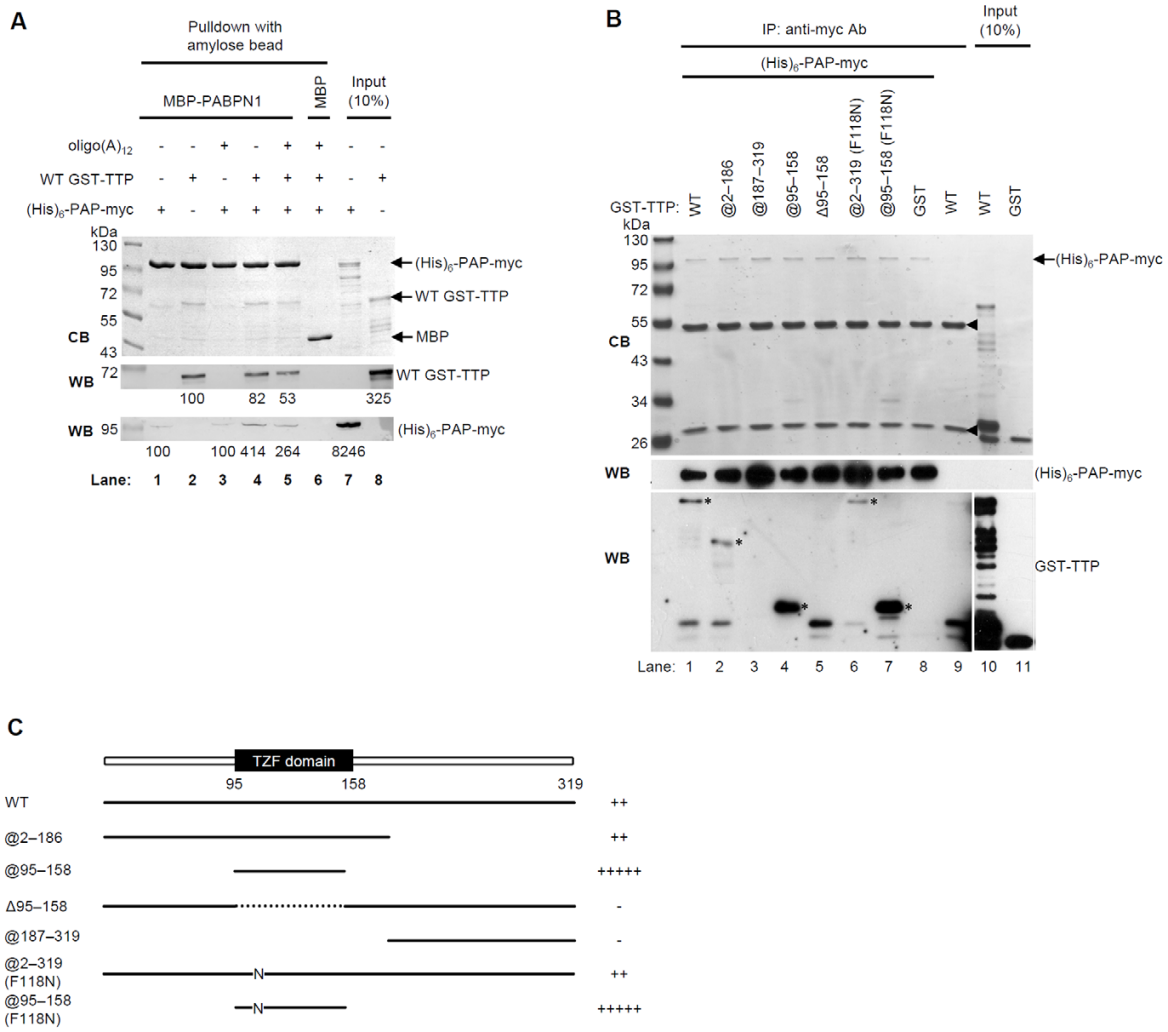
PAP interacts with TTP. (A) Pull-down assays to determine the interaction between MBP-PABPN1, (His)_6_-PAP-myc, and GST-TTP. MBP-PABPN1 was immobilized on amylose resin and then incubated with (His)_6_-PAP-myc (lane 1), or GST-TTP (lane 2), or both proteins (lanes 4–6). After extensive washes, the pulled-down complexes were subjected to SDS-PAGE, visualized with Coomassie Blue (CB, upper panel), and western blotted with anti-GST (WB, middle panel) or anti-myc (WB, lower panel). MBP (control) was incubated with (His)_6_-PAP-myc and GST-TTP (lane 6). Oligo(A)_12_ was added to the lane 3, 5, and 6 samples. The relative amounts of TTP and PAP visualized in the western blots were quantitated and indicated under the western blots. Lanes 7 and 8 correspond to samples that each contained 10% of the protein used for the pull-down experiments. (B) Co-immunoprecipitation assays for (His)_6_-PAP-myc and GST-TTP. GST-TTP mutants (0.5 µg) and (His)_6_-PAP-myc (0.5 µg) were co-immunoprecipitated with anti-myc resin. The precipitated proteins were resolved by SDS-PAGE, visualized with Coomassie Blue (CB, upper panel), and western blotted with anti-myc (WB, middle panel) or anti-GST antibodies (WB, lower panel). The RNA-binding deficient mutants GST-TTP@2-319 (F118N) and GST-TTP@95-158 (F118N) were also tested. Asterisks indicate the pulled-downed TTP constructs. The strong signals at ∼55 kDa and 26 kDa indicated with arrowheads in the upper gel are those of IgG heavy and light chains, respectively. (C) Schematics of the GST-TTP constructs used for the co-immunoprecipitation experiments of Fig. 4B. The relative amount of a TTP construct pulled down with PAP is indicated by the number of plus signs or, if not pulled down, by a negative sign. The included sequences are diagramed as heavy lines and the deleted regions are diagramed as dotted lines.

To determine if TTP competes with PAP for the PABPN1 arginine-rich region, we assessed if GST-TTP and MBP-PABPN1 could interact in the presence of hexahistidine ((His)_6_)-PAP-myc (Fig. 4A). MBP-PABPN1 was immobilized on amylose resin, and (His)_6_-PAP-myc and/or GST-TTP were the targets. The control sample used MBP as the bait, and (His)_6_-PAP-myc and GST-TTP as the targets (Fig. 4A, lane 6). PABPN1 weakly interacted with (His)_6_-PAP-myc (Fig. 4A, lane 1), which is consistent with a previous observation [27]. When both (His)_6_-PAP-myc and GST-TTP were present (Fig. 4A, lane 4) the amount of pull-downed TTP was ∼80% of that pulled down when it was present alone (Fig. 4A, lane 2); however, the amount of pull-downed (His)_6_-PAP-myc was four-fold greater than when present alone (Fig. 4A, lanes 1 and 4). Because PABPN1 binds poly(A), oligo(A)_12_ which contains twelve adenosines in the sequence was included in some of the pull-down systems. In the presence of oligo(A)_12_, the amount of pulled-down TTP decreased (Fig. 4A, lanes 4 and 5). Therefore, when bound to poly(A), the interaction between PABPN1 and TTP appears to be somewhat inhibited. Because the PABPN1 arginine-rich region interacts with TTP and PAP, TTP and PAP possibly compete for the PABPN1-binding site, causing less TTP to be pulled down; although, this explanation does not explain why more PAP was pulled down when TTP was present. We hypothesized that more PAP was pulled down in the presence of TTP because PAP can bind TTP, resulting in its co-precipitation with PABPN1. We used a co-immunoprecipitation assay to test if TTP interacts with PAP using several GST-TTP deletion constructs and (His)_6_-PAP-myc (Fig. 4B). Anti-myc resin was used to immunoprecipitate (His)_6_-PAP-myc. As controls, GST (Fig. 4B, lane 8) and no PAP (Fig. 4B, lane 9) were used to test for non-specific binding. The GST-TTP constructs containing the TZF domain WT GST-TTP, GST-TTP@2-186, and GST-TTP@95-158 (Fig. 4B, lane 1–2, and 4) co-immunoprecipitated with the PAP construct, and deletion of the TZF domain diminished the amount of TTP co-immunoprecipitated (Fig. 4B, lane 5). We therefore confirmed that TTP interacts with PAP via its TZF domain, which is also its RNA-binding domain [4]. Notably, the TZF construct bound more PAP than did the other TTP constructs. We also found that GST-TTP@2-319(F118N), which does not bind ARE [10], co-immunoprecipitated with PAP, as did GST-TTP@95-158(F118N) (Fig. 4B, lanes 6 and 7). The relative binding abilities of the WT and mutant GST-TTPs with PAP are diagramed in Fig. 4C as plus or minus signs. In summary, TTP directly interacted with PAP via its TZF domain; moreover, given the pull-down data, TTP seems to interact with PABPN1 and PAP simultaneously.

### TTP Inhibits Polyadenylation by PAP/PABPN1 of an ARE-containing RNA *in vitro*

**Figure 5:**
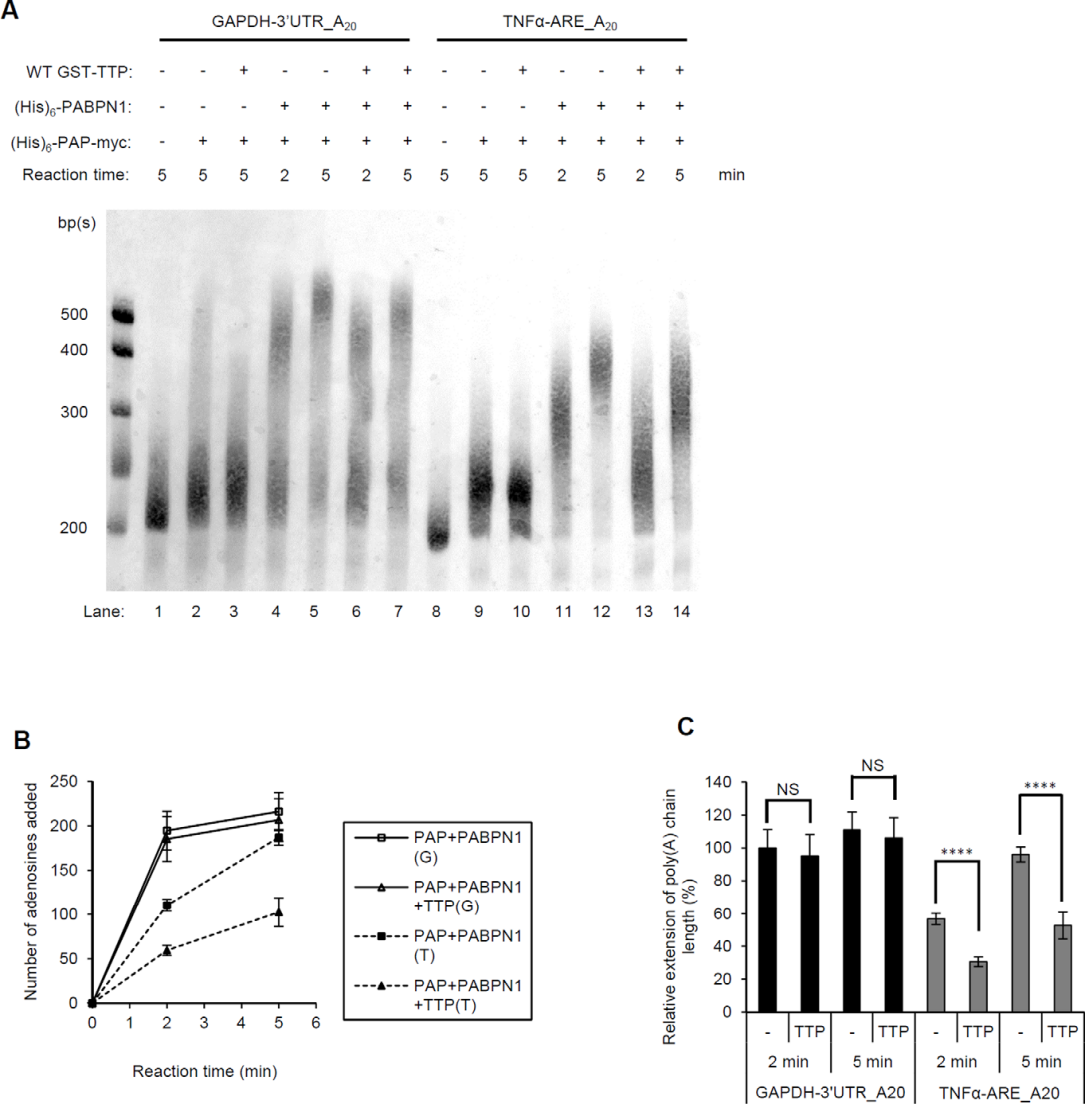
TTP inhibits PABPN1-stimulated PAP activity on ARE-containing mRNA. (A) Two biotinylated RNA templates were used for *in vitro* polyadenylation: an GAPDH mRNA that contained a partial 3'-UTR sequence and an TNFα mRNA that contained an ARE, both of which had 3′-20-mer poly(A) tails (GAPDH-3'-UTR_A_20_ and TNFα-ARE_A_20_, respectively). Purified (His)_6_-PAP-myc, (His)_6_-PABPN1, and GST-TTP were added into the reactions at 4°C, as indicated by a plus sign and reacted at 37°C for 2 or 5 min. RNA was separated through 8 M urea/5% (w/v) polyacrylamide gels, transferred to nylon membranes, and detected using horseradish peroxidase–labeled streptavidin. Each experiment was repeated four times, and a representative example is shown for each experiment. RNA molecular mass standards are shown in the left lane. (B) The migration distance of each RNA molecular mass standard vs. its number of base pairs was plotted and the plot was used to determine the lengths of the poly(A) tails by subtracting the size of the control RNA sample from the size of the experimental RNA sample. “G” and “T” in the boxed legend identify biotinylated GAPDH-3'UTR_A_20_ and TNFα-ARE_A_20_, respectively. (C) Quantification of the lengths of the poly(A) tails, normalized to the RNA GAPDH-3′UTR_A_20_ tail length found at 2 min. ****, p<0.001; ns, not significant.

CPSF and PABPN1 together promote polyadenylation by tethering PAP to pre-mRNA [26]. *In vitro* and in the absence of CPSF, PABPN1 facilitates the polyadenylation by PAP of oligo(A)-containing RNA. To further elucidate the function of the TTP/PABPN1 complex, we examined the effect of TTP on *in vitro* PAP/PABPN1 polyadenylation using biotinylated fragments of GAPDH mRNA containing its 3'-UTR, but no ARE (GAPDH-3'UTR_A_20_) and a TNFα-ARE-containing RNA (TNFα-ARE_A_20_), both of which contained 20-mer A tails. After polyadenylation by (His)_6_-PAP-myc, in the presence or absence of (His)_6_-PABPN1 and/or GST-TTP, RNA was resolved through urea/polyacrylamide gels, transferred to nylon membranes, and then detected with horseradish peroxidase–labeled streptavidin. The poly(A) tails of both RNA samples were distributively synthesized by PAP alone (Fig. 5A, lanes 2 and 9). The poly(A) tails increased by ∼200 and 100 As when PABPN1 was also present within 2 min (Fig. 5A, lanes 4 and 11, and Fig. 5B). The poly(A) tails then more gradually lengthened over the next 3 min (Fig. 5A, compare lanes 4 and 5, and 11 and 12, Fig. 5B). The poly(A) tails of GAPDH-3'UTR_A_20_ seemed to be longer than those of TNFα-ARE_A_20_ (Fig. 5B). The poly(A) tail length extensions shown in Fig. 5C were normalized to the average length of the GAPDH RNA sample at 2 min in the absence of TTP. Although synthesis of the GAPDH poly(A) RNA tail in the presence of PAP and PABPN1 seemed to be marginally decreased in the presence of TTP (Fig. 5A, compare lanes 4 and 6, and 5 and 7), the decrease was not statistically significant (Fig. 5C; ns). However, for TNFα-ARE_A_20_, TTP remarkably reduced the PABPN1-stimulated processive synthesis of the poly(A) tail (Fig. 5A, compare lanes 11 and 13, 12 and 14, and Fig. 5C; ****, p<0.001). The processive addition of adenosines to the TNFα RNA tail was reduced by ∼45% in the presence of TTP at both 2 and 5 min (Fig. 5C). The results of the RNA electrophoretic mobility shift assays (REMSA) also support this ARE-specific inhibition (Fig. S1). Even though we demonstrated a direct interaction between PABPN1 and TTP, the results of the REMSA indicated that TTP did not interact with the PABPN1/GAPDH-3′UTR_A_20_ complex. TTP did not bind to that complex (Fig. S1A), an observation that is consistent with the data presented in Fig. 4A, i.e., oligo(A)_12_ decreases association of PABPN1 and TTP. The decrease may be caused by a competition for the RNA-binding domain of PABPN1 by poly(A) and TTP. In the case of TNFα-ARE_A_20_, TTP and PABPN1 would bind to different regions, i.e., the ARE region and poly(A), respectively, which would allow a TTP/PABN1/TNFα-ARE_A_20_ complex to form (Fig. S1B). Interestingly, although only TTP could bind to TNFα-ARE (which did not contain a poly(A) tail), PABPN1 could then be recruited to form a larger complex, as shown by the antibody supershift assays that used anti-TTP and anti-PABPN1 (Fig. S1C, lanses 19 and 20).

**Figure 6:**
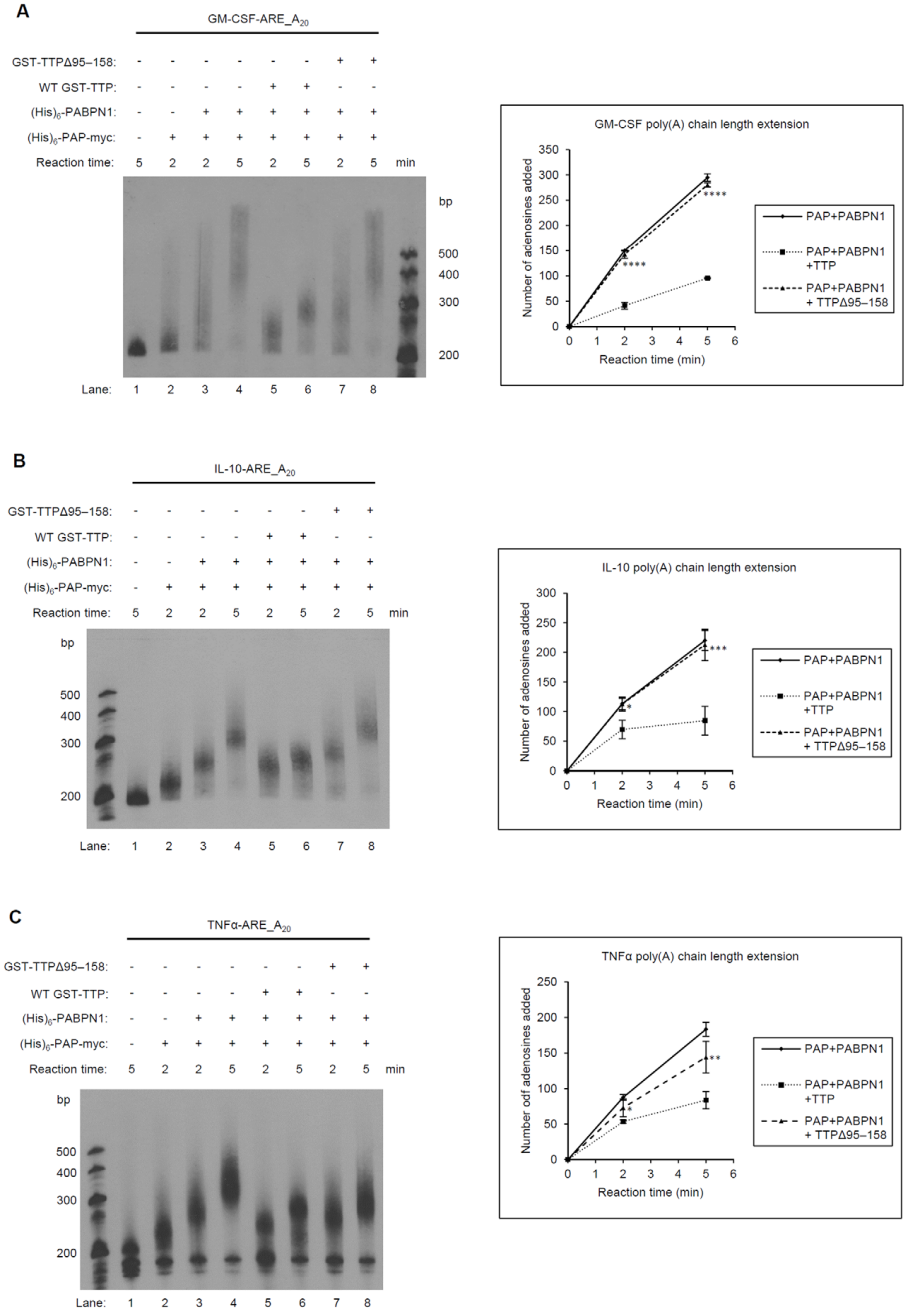
RNA-binding domain of TTP is required for *in vitro* polyadenylation inhibition in RNA containingARE. Biotin-labeled (A) GM- CSF-ARE_(A)_20_, (B) IL-10-ARE_(A)_20_, and (C) TNFα-ARE_(A)_20_ RNA were used for the *in vitro* polyadenylation assay as described in Fig. 5. The WT GST-TTP (lanes 5, 6) or GST-TTPΔ95-158 (lanes 7, 8) were included in the reaction mixtures. After reaction, RNA was separated through 8 M urea/8% (w/v) polyacrylamide gels. Each experiment was repeated three to five times, and a representative example is shown for each experiment. RNA molecular mass standards are shown in the left lane. The poly(A) tail lengths were calculated as described in Fig. 5 and plots of tail length vs. time are shown in the right panels. *, p<0.05; **, p<0.01; ***, p<0.005; ****, p<0.001.

To examine the global effect of TTP, other ARE-containing substrates, i.e., granulocyte-macrophage colony-stimulating factor (GM-CSF)-ARE_A_20_ and interleukin (IL)-10-ARE_A_20_, which can interact with TTP [30], [31], were also used for polyadenylation assays. TTP inhibited the polyadenylation of GM-CSF and IL-10 RNA (Fig. 6A, B). When GST-TTPΔ95–158 was substituted for WT GST-TTP, the levels of polyadenylation in the GM-CSF-ARE_A_20_, IL-10-ARE_A_20_, and TNFα-ARE_A_20_ increased (Fig. 6A–C, lanes 7, 8). The quantitative results were shown in the lower panels of Fig. 6A–C. Therefore, TTP inhibited the PAP/PABPN1 processive synthesis of RNA that contained the ARE region, and the TZF domain was critical for this effect.

### The TTP/PABPN1 Interaction Shortens the Poly(A) Tail of Nuclear TNFα mRNA

**Figure 7:**
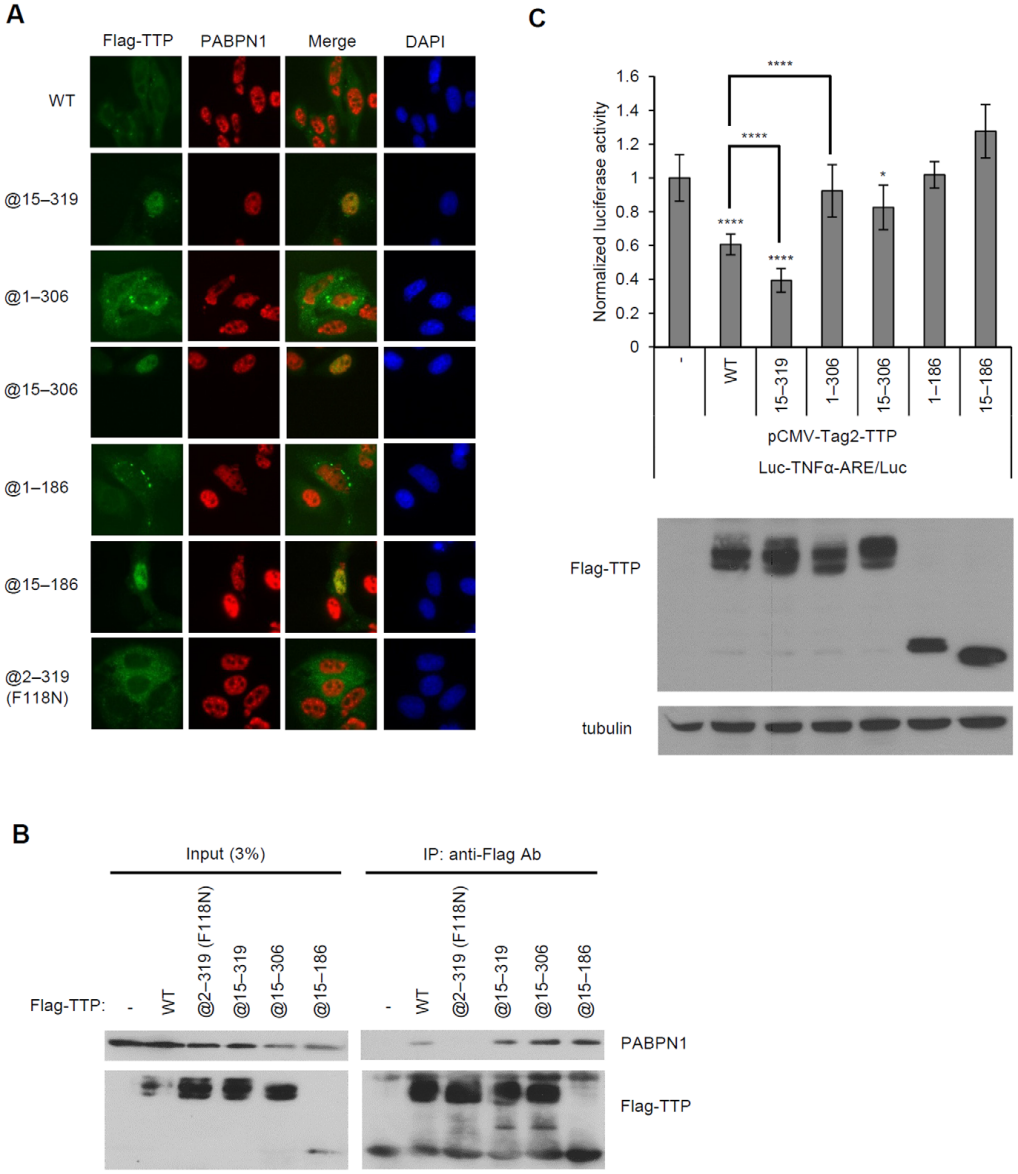
TTP functions in the nucleus. (A) Immunofluorescence staining. Plasmids encoding the Flag-TTP constructs listed in the figure were transfected into HeLa cells, and the protein products were immunostained with anti-Flag and anti-PABPN1. Flag-TTP constructs are stained green and endogenous PABPN1 is stained red. The blue signals are those of DAPI, which locate nuclei. (B) Co-immunoprecipitation of Flag-TTP constructs and PABPN1. HEK293T cells were transfected with plasmids encoding the indicated Flag-TTP constructs. After expression, the Flag constructs were immunoprecipitated from cell lysates with anti-Flag M2 agarose. Co-immunoprecipitated PABPN1 was detected by western blotting with anti-PABPN1. (C) Reporter assay using TNFα-ARE-containing luciferase plasmid. HEK293T cells were co-transfected with 0.5 µg of TNFα-ARE-containing luciferase plasmid or the control luciferase reporter plasmid and 0.1 µg of the WT Flag-TTP, 0.15 µg of Flag-TTP@15-319 or Flag-TTP@15-306 or Flag-TTP@15-186, 0.18 µg of Flag-TTP@1-306 and 0.22 µg of Flag-TTP@1-186 deletion mutant expression plasmid and 0.5 µg of the pRenilla-luciferase control plasmid. The protein expression levels were detected by western blotting with anti-Flag and the loading control anti-Tubulin in the lower panel. Duplicate reactions were each run three times. For each sample, its luciferase activity was first divided by its renilla luciferase activity and then normalized to the control firefly/renilla luciferase value. *, p<0.05; ****, p<0.001.

To explore the functional effect of the TTP/PABPN1 interaction in the nucleus and to rule out a destabilizing targeting effect by TTP in the cytoplasm, several Flag-tagged TTP deletion mutants were constructed and expressed in HeLa and HEK293T cells to obtain constructs that were retained in the nucleus. First, immunofluorescence staining was performed to monitor the subcellular locations of the TTP mutants (Fig. 7A). WT Flag-TTP was found mostly in the cytoplasm, whereas Flag-TTP@15-319, Flag-TTP@15-306, and Flag-TTP @15–186 remained mostly in the nucleus, which is a finding consistent with the suggestion that residues 1–14 may serve as a nuclear export signal [32]. Moreover, the co-immunoprecipitation results (Fig. 7B) for these Flag-TTP constructs with endogenous PABPN1 were consistent with those of the pull-down assays, i.e., the presence of the TZF domain and a region downstream to the residue 186 were sufficient for TTP and PABPN1 to interact. Interestingly, the functional reporter assay showed that nuclear dominant Flag-TTP@15-319 and Flag-TTP@15-306 still can suppress ARE-mediated luciferase activity, and Flag-TTP@15-319 displayed more suppressive extent than cytosol dominant WT Flag-TTP (Fig. 7C). Therefore, TTP may downregulate nuclear TNFα mRNA. Conversely, the mutants deleted in the C-terminal region did not have reporter-suppressing activity even when the TZF domain was present. Therefore, TTP may modulate nuclear gene expression only when its complete C-terminal region is present.

To determine if TTP regulates gene expression by interacting with PABPN1 and thereby reduces PAP activity, the poly(A) tail lengths of endogenous nuclear TNFα mRNA in LPS-stimulated RAW264.7 cells were characterized. After performing LM-PAT (Text S1 and Fig. S2A), the poly(A) tail lengths of nuclear TNFα mRNAs during LPS-stimulation were correlated with the TTP protein levels in nuclei (Fig. S2B). Recent reports have shown that TTP can recruit Caf1a to deadenylate and destabilize targeted ARE-containing mRNAs [12]–[14]. We detected Caf1a in the nuclei and cytoplasms (Fig. S2C). To rule out the possibility that a TTP-mediated deadenylation of nuclear TNFα mRNA occurred, Caf1a was knocked down using lentivirus carrying shCaf1a (Text S1). Compared with the shLuc samples, the amount of cytosolic TNFα mRNA increased in Caf1a knockdown cells during LPS stimulation (Fig. S2D). However, the profiles of the nuclear TNFα mRNA poly(A) tail lengths were the same for the control and the Caf1a-knockdown cell samples (Fig. S2E), suggesting a correlation between TTP expression and the shortening of the poly(A) tails in nuclear TNFα mRNA, which may be mainly caused by inhibition of polyadenylation rather than deadenylation.

## Discussion

By biopanning with a phage-display library, we found fragments from five different proteins that could bind TTP (Table S1). Biopanning with a phage-display library when searching for interacting proteins has certain advantages: 1) A library contains a highly diversified pool of ligands. 2) Biopanning can be performed under different reaction conditions, e.g., with different buffers. 3) The phage that interacts with the bait can be enriched by amplification in *E. coli* after biopanning. 4) Because a library consists of protein fragments, large complexes do not form as they do in co-immunoprecipitation assays.

Among the proteins identified by the biopan, we focused on the pre-mRNA processing protein PABPN1. PABPN1 promotes the polyadenylation activity of PAP and controls the length of poly(A) mRNA tails [26]. We demonstrated that TTP and PABPN1 interact with each other using *in vitro* pull-down assays with recombinant GST-TTP and MBP-PABPN1 constructs and *in vivo* co-immunoprecipitation assays. Many proteins have been identified as possible TTP targets when examined by immunoprecipitation or yeast two-hybrid screens [8], [10], [19], [33]–[36], but none of these proteins has been shown to directly interact with TTP.

By mapping their binding domains we found that the TZF and RRM domains of TTP and, the RNA-binding domain of PABPN1 are those that interact. Our *in vitro* pull-down assay showed that purified TTP and PABPN1 constructs interacted directly in the absence of RNA (Fig. 1D). After RNase treatment (Fig. 1C), the amount of TTP and PABPN1 co-immunoprecipitated from cellular extracts reflected the input protein levels, indicating their RNA-independent interaction in the cells. The results of the REMSA showed that TTP can bind mRNA ARE and PABPN1 simultaneously (Fig. S1). Therefore, TTP may bind to ARE via its TZF domain and interact with PABPN1 at the same time. Notably, the complete RRM and C-terminal arginine regions of PABPN1 are important for TTP binding. Supporting this result, TTP inhibited polyadenylation *in vitro* only when RNA contained an ARE (Figs. 5 and 6). TTP can bind ARE and inhibit the poly(A) tail synthesis via its TZF domain. Furthermore, because TTP can enhance mRNA deadenylation by associating with the Ccr4-Caf1-Not deadenylase complex [12]–[14], we knocked down Caf1a to exclude the possible TTP-mediated deadenylation. A shorter poly(A) tail also was observed that paralleled TTP expression (Fig. S2). Although this experiment did not fully rule out the possibility of deadenylation mediated by other deadenylases, it is one possible approach of separating deadenylation and inhibition of polyadenylation. These findings suggest that in nucleus, newly synthesized ARE-containing mRNA is recognized by TTP, which then interacts with PABPN1 to downregulate PAP activity.

There are five isoforms of poly(A) binding protein, and with the exception of PABPN1, they are primarily cytosolic poly(A)-binding proteins known as PABPCs [28], [37]. PABPC1 interacts with the poly(A) tails of cytosolic mRNA and eIF4G to enhance mRNA stability and promote translation. According to yeast two-hybrid screening and immunoprecipitation assays, PABPC1 and TTP bind [33], [35]. *In vitro* TTP-mediated mRNA deadenylation is inhibited by PABPC1. Both PABPN1 and PABPC1 shuttle between the nucleus and cytoplasm and function in both cell compartments [38]. The poly(A) tail of cap-binding protein heterodimers CBP80-CBP20-bound mRNA binds primarily PABPN1 and PABPC1; conversely, eIF4E-bound mRNA binds only PABPC1. PABPC1 replaces PABPN1 during the first round of translation [39]. In the fission yeast Pab2, an ortholog of mammalian PABPN1 is recruited to pre-mRNA early on during transcription and is retained on the translated mRNA [40]. PABPN1 is best known as a participant in mRNA polyadenylation as demonstrated by an *in vitro* biochemical study [28]. The RNA-destabilizing activity of TTP accelerates poly(A) tail removal [41], [42] and enhances degradation by associating mainly with the cytosolic RNA degradation machinery via its N-terminus [8], [10]. Interestingly, we detected the TTP/PABPN1 interaction only in nuclear extracts (Fig. 1C). TTP is usually located in the cytoplasm under steady-state conditions [32], [43], although it has occasionally been found primarily in the nucleus [15], [22]. The subcellular TTP distribution is affected by its phosphorylation state. MAPKAP kinase 2-phosphorylated TTP associates with the adaptor protein 14-3-3, which causes TTP to be directed to the cytoplasm in an inactive state and prevents the recruitment of cytoplasmic deadenylase [12], [13], [19]–[21], [44]. This finding suggests that active hypophosphorylated TTP might be located mostly in the nucleus [21]. We found by western blotting that nuclear TTP has a slightly greater mobility than does cytosolic TTP (Fig. 1C) and therefore suggest that the TTP/PABPN1 interaction may be regulated by TTP phosphorylation. We found that co-immunoprecipitation of TTP and PABPN1 was enhanced when p38 signaling had been inactivated (Fig. S3). Flag-TTP@15-319 was mostly retained in the nucleus and was capable of completely downregulating the expression of the luciferase reporter gene (Fig. 7C). Therefore, TTP may function in the nucleus. However, its exact function is unclear. We then found that the *in vitro* processive polyadenylation activity by PAP/PABPN1 was inhibited by TTP for only ARE-containing RNA (Fig. 6). Additionally, the poly(A) tail length of nuclear TNFα mRNA was shortened when the TTP expression level was increased in LPS-stimulated macrophages (Fig. S2). Our result seems to be consistent with a previous study that showed that a larger amount of TTP correlated with deadenylated TNFα mRNA accumulation [45]. Given our results, it is possible that the accumulation of deadenylated TNFα mRNA is caused by TTP shuttling into nucleus so as to affect polyadenylation.

Although residues 95–186 of TTP bound PABPN1 (Fig. 2), Flag-TTP@15-186 had no reporter-suppressing effect, which implicates the requirement for the C-terminal TTP region for full activity (Fig. 7C). The N-terminal domain of TTP is involved in mRNA degradation and does so by recruiting many different mRNA decay-related enzymes [8], [10]. However, optimal mRNA-destabilizing activity by TTP requires both the N-terminal and C-terminal domains [10], [46]. Because we found Flag-TTP@15-186 to be located primarily in the nucleus, TTP might perform its ARE-mediated degradation activity in the nucleus and involve its C-terminal region. Cth2, a TTP homolog in *S. cerevisiae*, has been reported to have at least two functions, one of which involves RNA 3'-end processing. Cth2 destabilizes ARE-containing mRNAs during iron starvation [47] and also affects poly(A) site selection by destabilizing extended transcripts produced by read-through processes [23]. We showed that TTP when interacting with PABPN1 inhibits processive poly(A) tail synthesis, thereby generating shorter-than-normal poly(A) tails. A previous report showed that influenza A virus NS1 protein interacts with PABPN1 to block the 3'-end processing of cellular pre-mRNAs [48]. The poly(A) tails of mature mRNAs are involved in mRNA export from the nucleus [49], mRNA stabilization [50], and translation efficiency [51]. In eukaryotic cells, the lack of a tail or one of insufficient length causes mRNA to be retained in nucleus [49]. In a PAP-deficient yeast strain, newly synthesized mRNA was degraded by nuclear exosomes [52]. Therefore, TTP-mediated defective polyadenylation in ARE-containing mRNA may direct mRNA to the nuclear surveillance pathway for degradation. The coupling of 3'-end processing and mRNA degradation by the TTP/PABPN1 complex to interfere with poly(A) tail synthesis is a new observation. We also partially characterized the nuclear function of TTP, which we will investigate further.

## Materials and Methods

### Cell Culture

[Thightest]Mouse macrophage RAW264.7 cells and HEK293T cells were purchased from ATCC and were grown at 37°C under a 5% CO_2_ atmosphere in Dulbecco's modified Eagle's medium (Gibco-BRL) supplemented with 10% fetal bovine serum (SAFC Biosciences or GIBCO Qualified), 100 U/ml penicillin, 0.1 mg/ml streptomycin (GIBCO).

### Phage-display Library Construction and Biopanning

RAW264.7 cells were first treated with 100 ng/ml LPS for 1 h. Total RNA from these cells was isolated using Ultraspec-II RNA Isolation System reagents (Biotecx) according to the manufacturer’s instructions. mRNA was extracted using Straight A’s mRNA Isolation System reagents (Novagen) according to the manufacturer’s instructions. cDNA was PCR synthesized and cloned into T7Select10-3b vectors in an orientation-specific manner using T7Select10-3 OrientExpress Random Primer cDNA Cloning System reagents (Novagen). Recombinant T7Select vectors were packaged into T7 vectors (T7 Packaging Extracts, Novagen) and propagated in *E. coli* Rosetta-gami B 5615 (Novagen). For each biopanning, 10 µg of the bait protein, MBP-TTP-(His)_6_, was immobilized on 20 µl of Ni-NTA resin (Qiagen). Unbound bait was washed with 50 mM Tris-Cl, pH 7.5, 150 mM NaCl, 0.1% (v/v) Tween-20. Then the amplified phage library was added and incubated overnight at 4°C. The resin was washed with 50 mM Tris-Cl, pH 7.5, 150 mM NaCl, 0.1% (v/v) Tween-20 to remove unbound phage. Bound phages were eluted from the Ni-NTA or amylose resin with 200 mM imidazole or 10 mM maltose in 50 mM Tris-Cl, pH 7.5, 150 mM NaCl, 0.1% (v/v) Tween-20, respectively. Eluted phage were individually amplified in 1 ml of a log-phase *E. coli* Rosetta-gami B 5615 cell lysate at 37°C until the lysate cleared. After centrifugation, the supernatant was biopanned again. Amplification and biopanning were repeated for 10 rounds. The fourth, ninth, and tenth rounds were titrated so that a single plaque could be added into 20 mM Tris, pH 8.0, 100 mM NaCl, 6 mM MgSO_4_ for amplification. For phage recovered from the Ni-NTA resin, 24 from the fourth round, 144 from the ninth round, and 240 from the tenth round were picked. The phage inserts were PCR amplified using T7Select primers, their sequences were determined, and their correct reading frames were confirmed by comparison with those in the NCBI databases using BLAST. One of the sequences contained nucleotides 404 to 922 of PABPN1 [NM_019403].

### Plasmid Constructs

Mouse WT TTP cDNA [NM_011756] was PCR amplified using template cDNA from LPS-treated RAW264.7 cells. For biopanning, WT TTP cDNA was PCR amplified using primers containing *Eco*RI and *Hind*III restriction sites and a (His)_6_-tag oligonucleotide sequence incorporated into the downstream primer. The PCR product was cloned into a pMAL-c4x bacteria expression vector (New England Biolabs) that was predigested with EcoRI and HindIII. For pull-down assays, WT and TTP nucleotide sequences containing deletions were PCR amplified using primers containing *Eco*RI and *Hind*III restriction sites, and then each cloned into a pGEX-4T-1 bacterial expression vector (GE Healthcare) to produce a GST-tagged construct. Each TTP cDNA construct was also inserted into a pCMV-Tag2 (Stratagene) vector to produce an N-terminally Flag-tagged protein. Additionally, a pMAL-c4x vector was modified by addition of the tandem affinity hemagglutinin (HA)-(His)_6_ tag at the downstream end of the gene to produce a pMAL-HA-(His)_6_ expression vector. PCR-amplified PABPN1 nucleotide sequences containing deletions were individually cloned into a pMAL-HA-(His)_6_ vector that contained *BamH*I and *Sal*I restriction sites. The genes for PAP [NM_011112] and its deletion mutants were each inserted into a pQE expression vector, and nucleotide sequences for (His)_6_ and myc tags were incorporated into the upstream and downstream ends, respectively.

### Recombinant Protein Expression and Purification

Recombinant proteins were expressed in *E. coli* STBL2 or Rosetta(DE3)pLacI cells. MBP-TTP-(His)_6_, MBP-PABPN1-HA-(His)_6_, and their deletion mutants were purified by Ni-NTA chromatography. GST-TTP and its deletion mutants were purified using glutathione-agarose resin (Sigma-Aldrich).

### Pull-down Assays

Purified MBP-PABPN1-HA-(His)_6_ or one of its deletion mutants (1 µg each) was used as bait after immobilization on amylose resin. Unbound bait was removed with 20 mM Tris-Cl, pH 7.0, 150 mM NaCl, 0.1% (v/v) NP-40, 5 mM MgCl_2_, 1 mM dithiothreitol (DTT). Then, 1 µg of a target protein was added and incubated overnight at 4°C. After removing unbound target with 20 mM Tris-Cl, pH 7.0, 150 mM NaCl, 0.1% (v/v) NP-40, 5 mM MgCl_2_, 1 mM DTT, the resin was suspended in SDS-PAGE loading buffer and boiled to elute the bait and target. The eluted proteins were separated by SDS-PAGE, Coomassie Blue visualized, and then were western blotted and probed with anti-GST antibodies and horseradish peroxidase–labeled secondary antibodies prior to diaminobenzidine staining or enhanced chemiluminescence (GE Healthcare).

### Cytosolic, Nuclear, and Whole-cell Extract Preparations and Immunoprecipitation Assays

Cells were harvested 24 h after transfection and then lysed in 10 mM HEPES, pH 7.4, 10 mM potassium acetate, 2.5 mM DTT, 1.5 mM MgCl_2_, 0.05% (v/v) NP-40 20% (v/v) glycerol, and cocktails of protease (Sigma-Aldrich) and phosphatase inhibitors including 0.01 M β-glycerol phosphate, 0.1 mM Na_2_MoO_4_, 0.1 mM Na_3_VO_4_ and 0.01M NaF. For RNase treatment, the RNase cocktail was 1 µg/ml RNase A (Amresco) and 10 U/ml RNase T1 (Sigma-Aldrich) (final concentrations). After incubation on ice for 15 min, the cell lysates were centrifuged, and the supernatants were used as the cytosolic extracts. Then, each pellet was suspended in 20 mM HEPES, pH 7.4, 400 mM NaCl, 1 mM EDTA, 1 mM EGTA, 1 mM DTT, 20% (v/v) glycerol, vigorously vortexed for 15 min at 4°C, and centrifuged. The supernatants were retained as the nuclear extracts. The concentration of NaCl in the nuclear extracts was diluted to 100 mM, and then the cytosolic and nuclear extracts were precleaned using protein-A agarose (Sigma-Aldrich) for 1 h at 4°C. The cleaned supernatants were immunoprecipitated with anti-Flag M2 affinity agarose (Sigma-Aldrich) for 1 h at 4°C.

For endogenous protein immunoprecipitation, RAW264.7 cells were treated with 100 ng/ml LPS for 6 h to induce TTP expression. Whole cellular protein was extracted with 25 mM HEPES, pH 7.5, 300 mM NaCl, 1.5 mM MgCl_2_, 0.2 mM EDTA, 0.1% (v/v) TritonX-100, 20% (v/v) glycerol, and the aforementioned protease inhibitor cocktail. After extraction, the NaCl concentration was adjusted to 150 mM. Next, the lysates were treated with calf intestinal phosphatase (New England Biolabs) for 1 h at 37°C to obtain hypophosphorylated TTP and then cleaned with protein A Sepharose (Sigma-Aldrich). Immunoprecipitation was then performed with anti-PABPN1 (Abcam) at 4°C for 3 h. Unbound proteins were removed, and the co-immunoprecipitates were detected with anti-TTP. To detect recombinant protein interactions, anti-myc agarose (Clontech) was incubated with 0.5 µg of bacterially expressed GST-TTP and (His)_6_-PAP-myc in 10 mM Tris, pH 7.0, 0.15 M NaCl, 1 mM MgCl_2_, 0.7 mM ZnCl_2_, and 0.1% (v/v) NP-40 at 4°C overnight. After washing, the immunoprecipitates were resolved by SDS-PAGE, and their contents identified by western blotting with anti-Myc and anti-GST (Sigma-Aldrich).

### *In vitro* Polyadenylation Assay

The DNA fragments used for synthesis of the RNA substrates were obtained by PCR using the following primer sequences that contained the T7 promoter (TAATACGACTCACTATAGG) at the 5′ end and 20 thymidines (T)_20_ at the 3′ end : 5′-(T7 promoter)-TGAGGTGCAATGCACAGC-3′ and 5′-(T)_20_-CCGGCCTTCCAAATAAATAC-3′ for *TNFα*; 5′-(T7 promoter)-TTGGACAGCGGAAGACA-3′ and 5′-(T)_20_- AAAAGTTTTAATAATTTA-3′ for *GM-CSF* (NM_009969.4); 5′-(T7 promoter)-ATTTATTACCTCTGATAC-3′ and 5′-(T)_20_- CCTTTAAATACTATAAAG-3′ for *IL-10* (NM_010548.2); 5′-(T7 promoter)- TCTCCCTCACAATTTCCA-3′ and 5′-(T)_20_-GGGTGCAGCGAACTTTAT-3′ for *GAPDH*. The mRNA templates used in the polyadenylation assays were synthesized *in vitro* with T7 RNA polymerase using Megashortscript kit reagents (Ambion) and then biotinylated with biotin-CTP (Invitrogen) according to the manufacturer’s instructions. The polyadenylation assays were performed as described [26], except that 0.5 µM ZnCl_2_ was included in the reaction buffer. For the polyadenylation reactions, 500 fmol of an RNA template, 50 fmol PAP, 5 pmol PABPN1, and 1 pmol TTP were included. Total reaction volume was 20 µl. All reaction components were held on ice before initiating the reaction, which was started by increasing the temperature to 37°C and stopped by immersion in liquid nitrogen. Proteins were then digested with proteinase K [25]. The RNA templates were precipitated with ethanol, resolved through 8 M urea/5% (w/v) polyacrylamide gels, and then transferred to nylon membranes using the SemiPhor Semi-Dry Transfer Unit with TBE (Tris/Borate/EDTA). The membranes were blocked with 1% (w/v) SDS and 3% (w/v) BSA in phosphate-buffered saline. Horseradish peroxidase–labeled streptavidin (Sigma-Aldrich) was used for hybridization with the biotin-labeled RNA and the RNA signal was detected with diaminobenzidine or with Western lightening- enhanced chemiluminescence substrate (PerkinElmer).

### Immunofluorescence Staining

HeLa cells were transfected using Lipofectamine 2000 (Invitrogen). Cells were subjected to immunofluorescence staining 24 h after transfection as described [53]. The primary antibody was mouse anti-Flag (Sigma-Aldrich) or rabbit anti-PABPN1 (Abcam). The secondary antibody was Alexa Fluor 594–conjugated goat anti-rabbit IgG or Alexa Fluor 488–conjugated goat anti-mouse IgG, as appropriate. To visualize the nuclei, cells were stained with 4', 6-diamidino-2-phenylindole (DAPI) during the secondary antibody incubation. The locations of the Flag-TTP constructs and endogenous PABPN1 were visualized using a Leica DM6000 B Upright Microscope.

### Transfection and Luciferase-reporter Assay

HEK293T cells were transfected by the calcium phosphate precipitation method with 0.5 µg of a TNFα ARE**/**luciferase-reporter plasmid [54] or a control luciferase-reporter plasmid, 0.5 µg of the pRenilla-luciferase control plasmid, and 0.1 µg of the WT Flag-TTP, 0.15 µg of Flag-TTP@15-319 or Flag-TTP@15-306 or Flag-TTP@15-186, 0.18 µg of Flag-TTP@1-306 and 0.22 µg of Flag-TTP@1-186 deletion mutant expression plasmid. The luciferase assay was performed as technical manual of dual luciferase reporter assay system (Promega).

### Statistical Analysis

All of the data are presented as the mean ± SD of at least three independent experiments. The statistically significant values were determined by two-tailed Student's *t*-test.

## Supporting Information

Figure S1 **TTP and PABPN1 form a complex on ARE-containing mRNA.** DNA templates for GAPDH-3'UTR_A_20_, TNFα-ARE_A_20_, and TNFα-ARE RNA were PCR synthesized as described in Materials and Methods. REMSA assays were performed as previously described [54] by incubating ^32^P-labeled (A) GAPDH-3'UTR_A_20_, (B) TNFα-ARE_A_20_, or (C) TNFα-ARE with WT GST-TTP (10 ng), and/or (His)_6_-PABPN1 (10- to 20-fold excess), and/or (His)_6_-PAP-myc (10- to 20-fold excess), and antibodies as indicated above the lanes. BSA served as the negative control in (C). The RNA-protein complexes were resolved by native polyacrylamide gel electrophoresis and then subjected to autoradiography. The asterisk indicates a signal in the probe TNFα-ARE; and the arrowheads indicate the supershift signals by anti-TTP and anti-PABPN1. Each experiment was repeated three to five times, with a representative result displayed.
(TIF)

Figure S2 **TTP expression affects the poly(A) tail length in TNFα mRNA from LPS-stimulated RAW264.7 cells.** (A) Schematic of the LM-PAT method. (B) The expression level and poly(A) tail length of nuclear TNFα mRNA during LPS stimulation. RAW264.7 cells were treated with 100 ng/ml LPS for 0, 15, 30, 60, or 120 min. Nuclear RNA was then isolated to determine the TNFα mRNA poly(A) tail length. The cytoplasmic and nuclear extracts were isolated for western blotting with anti-TTP. Anti-tubulin and anti-hnRNP C1/C2 served as controls for the cytoplasmic and nuclear fractions, respectively. (C) Distribution of Caf1a in RAW264.7 cells. An equal quantity of cytosolic and nuclear protein was subjected to SDS-PAGE. The location of Caf1a was detected by western blotting with anti-Caf1a. Tubulin and hnRNP C1/C2 served as the internal cytosolic and nuclear controls, respectively. (D) Cytosolic TNFα mRNA activation profile in RAW264.7 control and cells expressing shCaf1a. After stimulation for 0, 15, 30, 60, or 120 min, cytosolic RNA from the Caf1a-knockdown and control RAW264.7 cells was isolated. The cytosolic TNFα mRNA fold activation was determined by quantitative PCR. **, p<0.01; ****, p<0.001. (E) The poly(A) tail length of nuclear TNFα mRNA during LPS stimulation in Caf1a-knockdown or control RAW264.7 cells. RAW264.7 cells were treated with 100 ng/ml LPS for 0, 15, 30, 60, or 120 min. Nuclear RNA was isolated for LM-PAT to determine the TNFα mRNA poly(A) tail length (upper left panel). The knockdown efficiency of shCaf1a for the cytoplasmic and nuclear TTP-expression profiles in the shCaf1a-expressed RAW264.7 cells is shown in the lower panel.
(TIF)

Figure S3 **PABPN1 interacts with only hypophosphorylated TTP.** HEK293T cells were cotransfected with a Flag-TTP expression plasmid, a Flag-p38 expression plasmid, and plasmids for constitutively active (CA) or dominantly negative (DN) Flag-MKK3. Whole-cell extracts were isolated and immunoprecipitated with anti-Flag M2 agarose. The immunoprecipitates were western blotted with anti-PABPN1 and anti-TTP. Expression of the DN Flag-MKK3 resulted in a hypophosphorylated TTP of lesser molecular mass that complexed with PABPN1, whereas hyperphosphorylated TTP did not interact with PABPN1.
(TIF)

Table S1 **Proteins corresponding to the sequences retrieved by biopanning with TTP.**
(TIF)

Text S1 **Supplemental materials and methods:** Ligation-mediated poly(A) test (LM-PAT) [55] and Lentivirus-mediated knockdown of Caf1a.
(DOCX)
