# Supplementary figures and images for: Tristetraprolin Inhibits Poly(A)-Tail Synthesis in Nuclear mRNA that Contains AU-Rich Elements by Interacting with Poly(A)-Binding Protein Nuclear 1
Source: PLoS One. 2012 Jul 26;7(7):e41313. doi: 10.1371/journal.pone.0041313 (PMC3406032; doi:10.1371/journal.pone.0041313)

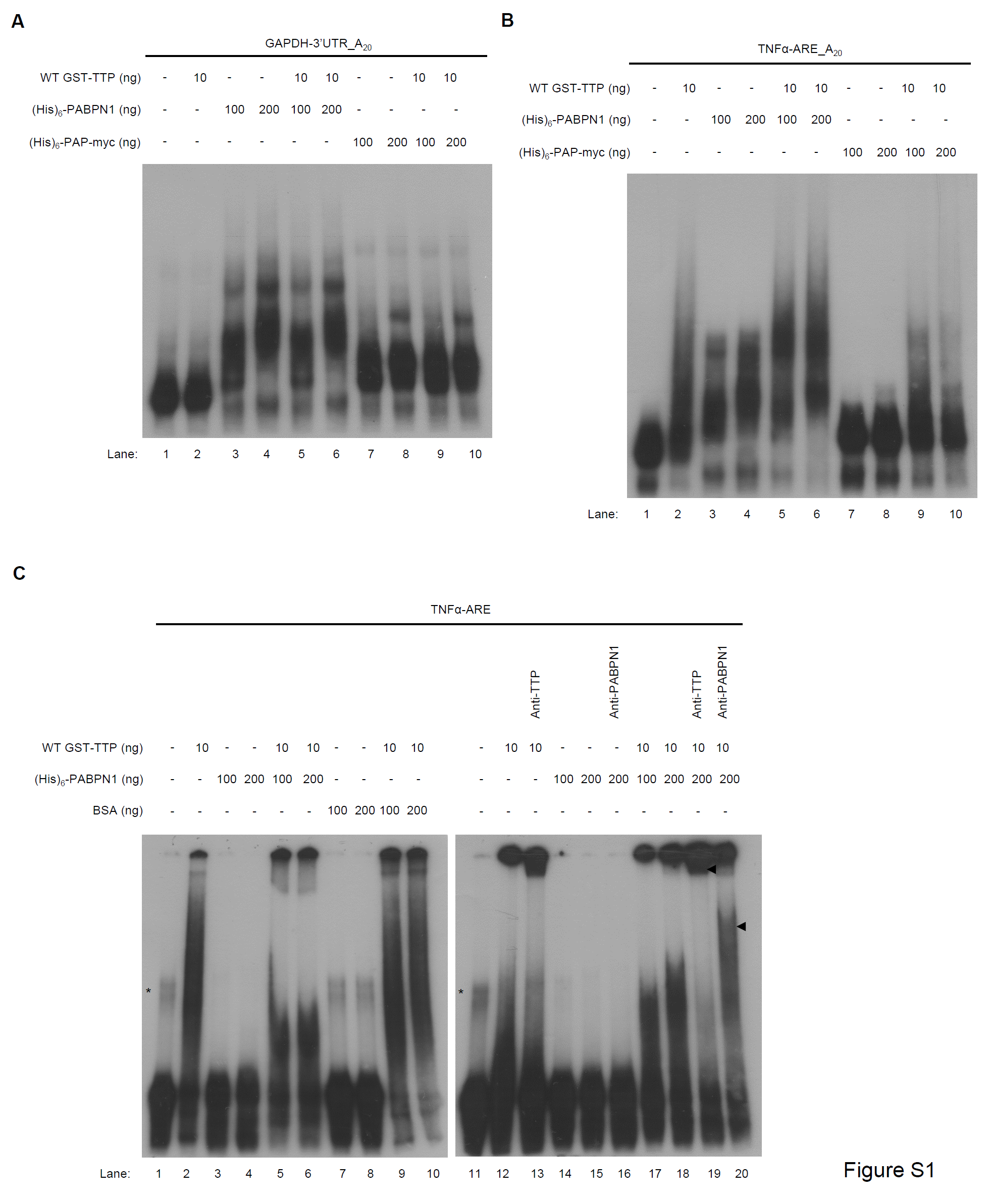

Supplement: Figure S1 — TTP and PABPN1 form a complex on ARE-containing mRNA. DNA templates for GAPDH-3'UTR_A20, TNFα-ARE_A20, and TNFα-ARE RNA were PCR synthesized as described in Materials and Methods. REMSA assays were performed as previously described [54] by incubating 32P-labeled (A) GAPDH-3'UTR_A20, (B) TNFα-ARE_A20, or (C) TNFα-ARE with WT GST-TTP (10 ng), and/or (His)6-PABPN1 (10- to 20-fold excess), and/or (His)6-PAP-myc (10- to 20-fold excess), and antibodies as indicated above the lanes. BSA served as the negative control in (C). The RNA-protein complexes were resolved by native polyacrylamide gel electrophoresis and then subjected to autoradiography. The asterisk indicates a signal in the probe TNFα-ARE; and the arrowheads indicate the supershift signals by anti-TTP and anti-PABPN1. Each experiment was repeated three to five times, with a representative result displayed. (TIF) [file pone.0041313.s001.tif]

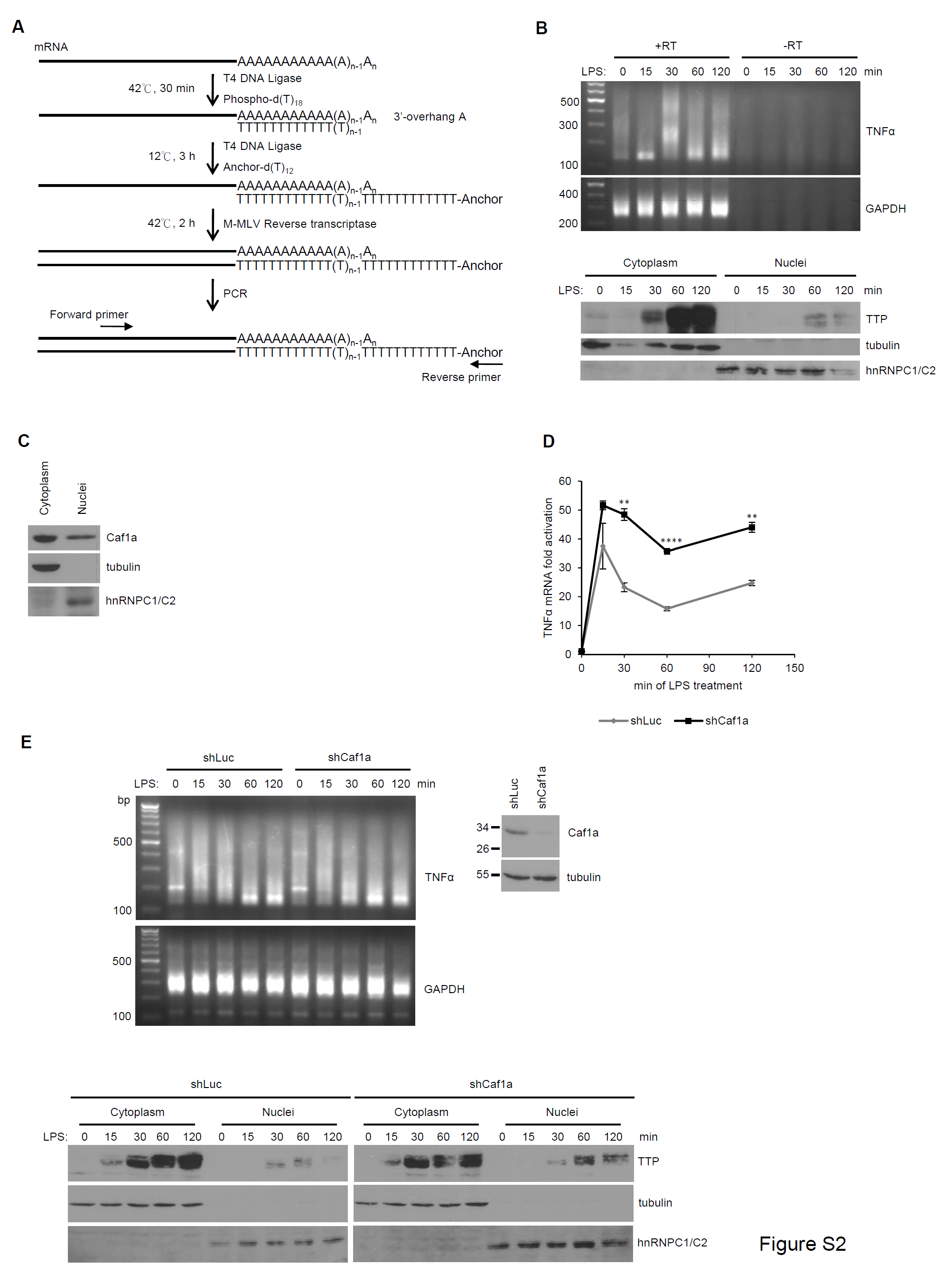

Supplement: Figure S2 — TTP expression affects the poly(A) tail length in TNFα mRNA from LPS-stimulated RAW264.7 cells. (A) Schematic of the LM-PAT method. (B) The expression level and poly(A) tail length of nuclear TNFα mRNA during LPS stimulation. RAW264.7 cells were treated with 100 ng/ml LPS for 0, 15, 30, 60, or 120 min. Nuclear RNA was then isolated to determine the TNFα mRNA poly(A) tail length. The cytoplasmic and nuclear extracts were isolated for western blotting with anti-TTP. Anti-tubulin and anti-hnRNP C1/C2 served as controls for the cytoplasmic and nuclear fractions, respectively. (C) Distribution of Caf1a in RAW264.7 cells. An equal quantity of cytosolic and nuclear protein was subjected to SDS-PAGE. The location of Caf1a was detected by western blotting with anti-Caf1a. Tubulin and hnRNP C1/C2 served as the internal cytosolic and nuclear controls, respectively. (D) Cytosolic TNFα mRNA activation profile in RAW264.7 control and cells expressing shCaf1a. After stimulation for 0, 15, 30, 60, or 120 min, cytosolic RNA from the Caf1a-knockdown and control RAW264.7 cells was isolated. The cytosolic TNFα mRNA fold activation was determined by quantitative PCR. **, p<0.01; ****, p<0.001. (E) The poly(A) tail length of nuclear TNFα mRNA during LPS stimulation in Caf1a-knockdown or control RAW264.7 cells. RAW264.7 cells were treated with 100 ng/ml LPS for 0, 15, 30, 60, or 120 min. Nuclear RNA was isolated for LM-PAT to determine the TNFα mRNA poly(A) tail length (upper left panel). The knockdown efficiency of shCaf1a for the cytoplasmic and nuclear TTP-expression profiles in the shCaf1a-expressed RAW264.7 cells is shown in the lower panel. (TIF) [file pone.0041313.s002.tif]

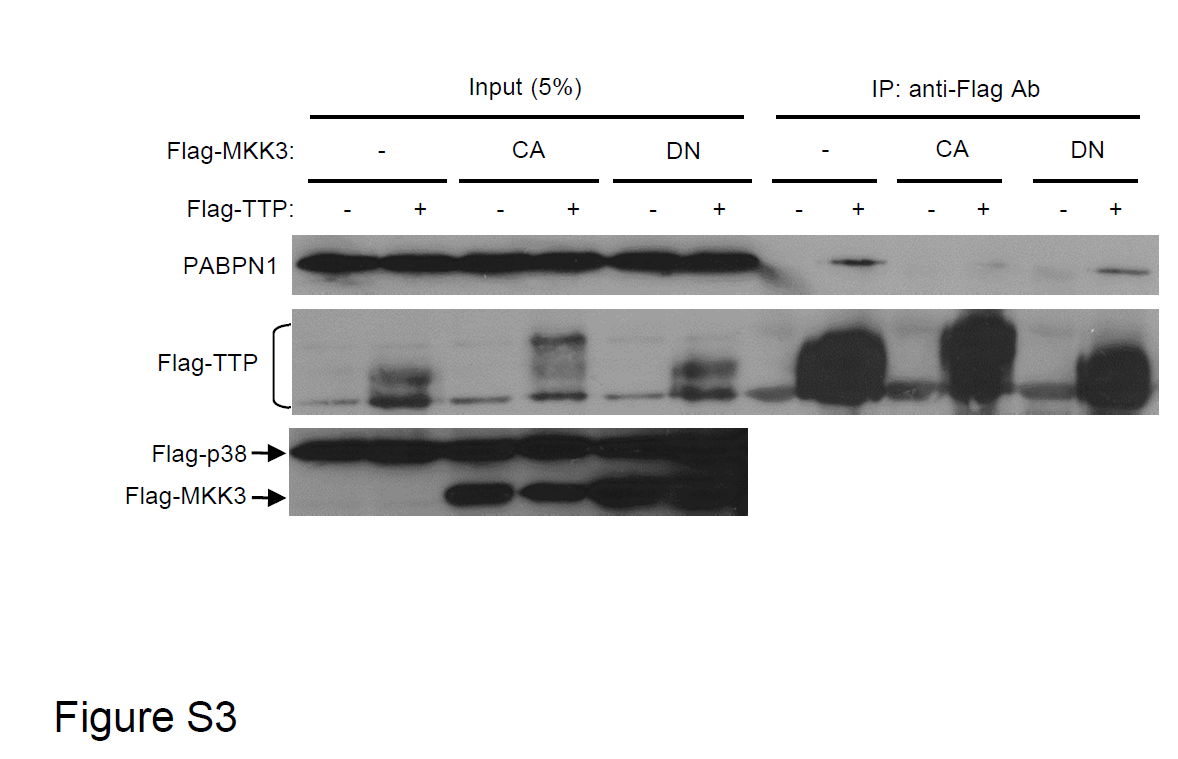

Supplement: Figure S3 — PABPN1 interacts with only hypophosphorylated TTP. HEK293T cells were cotransfected with a Flag-TTP expression plasmid, a Flag-p38 expression plasmid, and plasmids for constitutively active (CA) or dominantly negative (DN) Flag-MKK3. Whole-cell extracts were isolated and immunoprecipitated with anti-Flag M2 agarose. The immunoprecipitates were western blotted with anti-PABPN1 and anti-TTP. Expression of the DN Flag-MKK3 resulted in a hypophosphorylated TTP of lesser molecular mass that complexed with PABPN1, whereas hyperphosphorylated TTP did not interact with PABPN1. (TIF) [file pone.0041313.s003.tif]

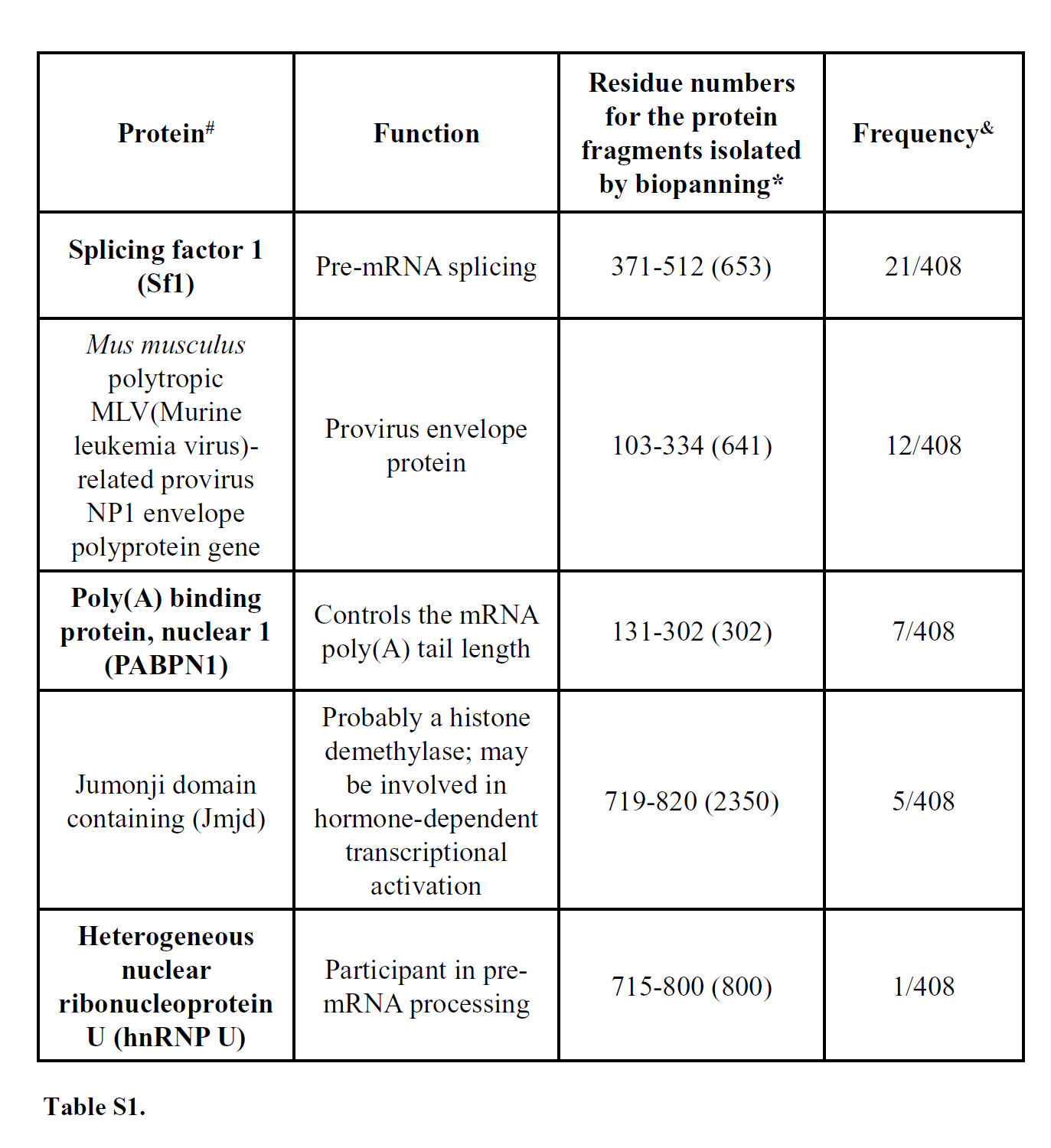

Supplement: Table S1 — Proteins corresponding to the sequences retrieved by biopanning with TTP. (TIF) [file pone.0041313.s004.tif]
