# Supplementary material for: Tristetraprolin Inhibits Poly(A)-Tail Synthesis in Nuclear mRNA that Contains AU-Rich Elements by Interacting with Poly(A)-Binding Protein Nuclear 1
Source: PLoS One. 2012 Jul 26;7(7):e41313. doi: 10.1371/journal.pone.0041313 (PMC3406032; doi:10.1371/journal.pone.0041313)
Supplement: Text S1 — Supplemental materials and methods: Ligation-mediated poly(A) test (LM-PAT) [55] and Lentivirus-mediated knockdown of Caf1a. (DOCX) [file pone.0041313.s005.docx]

Text S1. Supplemental Materials and Methods

*Ligation-mediated poly(A) test (LM-PAT)*

Total RNA was extracted using TRIzol (Invitrogen) from RAW264.7 cells that had been treated with LPS for different lengths of time. Before the LM-PAT, RNA samples were digested with Turbo DNA-free reagent (Ambion) to remove genomic DNA and then extracted again with TRIzol. The LM-PAT was performed as previously described. The resulting cDNA was amplified by semi-quantitative PCR with the forward primer 5'-GGTGACCAGGCTGTCGCTACATC-3' for *TNFα* or 5'-GGTGGACCTCATGGCCTACATGG-3' for *GAPDH*. To characterize the lengths of the poly(A) tails, the anchor sequence 5'-GCGAGCTCCGCGGCCGCG-3' served as the reverse primer. The PCR cycling conditions were: 5 min at 95°C; 30 s at 95°C, 30 s at 58°C, and 1 min at 72°C for 35 cycles; then 5 min at 72°C. PCR products were characterized after separation through a 2% (w/v) agarose gel.

*Lentivirus-mediated knockdown of Caf1a*

Lentivirus carrying pLKO.1-shRNA was produced in 293T cells transfected with pCMVΔ8.91, pMD.G, and pLKO.1-shRNA. Mouse shCaf1a and the control shLuc, which targeted *Luciferase* were purchased from the National RNAi Core Facility of Academia Sinica (Taipei, Taiwan) The sequence of shCaf1a is 5'-CCGGGCGGTGTAATGTAGACTTGTTCTCGAGAACAAGTCTACATTACACCGCTTTTTG-3'. RAW264.7 cells were infected with virus for 48 h, and then infected colonies were selected with puromycin. The knockdown efficiency of shCaf1a was confirmed by probing with anti-Caf1a antibodies (Abnova). Cells that stably expressed shRNA were treated with 100 ng/ml LPS for different periods of time. Cytosolic and nuclear RNA were isolated for LM-PAT and reverse transcription. Real-time PCR was performed to validate the cytosolic quantity of TNFα mRNA.
